# Semen characteristics, extension, and cryopreservation of Rusa deer (*Rusa timorensis)*

**DOI:** 10.14202/vetworld.2017.779-785

**Published:** 2017-07-14

**Authors:** Wan-Nor Fitri, Haron Wahid, Yusoff Rosnina, Faez Firdaus Abdullah Jesse, Zainal Abidin Aimi-Sarah, Mohd Lila Mohd-Azmi, Che’ Amat Azlan, Muhammad Rashid Azrolharith, Innocent Damudu Peter, Falah Hasan Ali Baiee

**Affiliations:** 1 Department of Veterinary Clinical Studies, Universiti Putra Malaysia, 43400 UPM, Serdang, Selangor, Malaysia; 2 Research Centre for Wildlife, Faculty of Veterinary Medicine, Universiti Putra Malaysia, 43400 UPM, Serdang, Selangor, Malaysia; 3 Research Centre for Ruminant Diseases, Faculty of Veterinary Medicine, Universiti Putra Malaysia, 43400 UPM, Serdang, Selangor, Malaysia; 4 Food Science and Technology Program School of Industrial Technology, Faculty of Applied Science, Universiti Teknologi Mara (UiTM), 40450 Shah Alam, Selangor, Malaysia; 5 Department of Veterinary Pathology and Microbiology, Universiti Putra Malaysia, 43400 UPM, Serdang, Selangor, Malaysia; 6 Department of Medicine & Surgery of Farm & Exotic Animal, Faculty of Veterinary Medicine, Universiti Putra Malaysia, 43400 UPM, Serdang, Selangor, Malaysia

**Keywords:** cryopreservation, electroejaculation, extension, Rusa deer, semen characteristics

## Abstract

**Aim:**

The objective of this research is to report parameters for breeding soundness evaluation, semen extension, and cryopreservation in *Rusa timorensis*.

**Materials and Methods:**

Seven healthy stags were chosen for semen collection using an electroejaculator. The collections were performed twice in a breeding season between February and June 2016. Samples were collected between 2 and 3 weeks interval, collected twice for each animal. Semen was evaluated, extended, and cryopreserved using four different extenders; Andromed^®^, BioXcell^®^, Triladyl^®^, and a modified Tris-egg yolk combined with *Eurycoma longifolia* Jack.

**Results:**

*R. timorensis* semen characteristics according to volume (ml), color, sperm concentration (10^6^/ml), general motility (%), progressive motility (%), and % morphology of normal spermatozoa are 0.86±0.18 ml, thin milky to milky, 1194.2±346.1 10^6^/ml, 82.9±2.8%, 76.1±4.8%, and 83.9±4.8%, respectively.

**Conclusion:**

Semen characteristics of *R. timorensis* collected by electroejaculation is good allowing for cryopreservation and future artificial insemination work. The most suitable extender for Rusa deer semen is Andromed^®^.

## Introduction

Spermatozoa had long been the center of study to humankind to bring forth the gift of life in the form of semen. Semen characteristics are usually used as indicators of reproductive soundness of study [1]. However, these parameters are not well established in Rusa deer (*Rusa timorensis*). A widely accepted method for safe semen collection in untrained animal is using an electroejaculator under general anesthesia [2]. This method yields acceptable semen quality in most cases. Study in semen has also led to early pioneering study of artificial insemination (AI) in the veterinary field [3]. Other study in domesticated wildlife species has been shown possible with acceptable semen quality for insemination [4–6]. Modern day science of animal reproduction has long advanced since then accompanied with the development of technology in domestic animals [7-9]. The number of hinds for AI, mostly red deer, only account for 1% of national breeding population in New Zealand [10]. In contrast, 20% of cows bred worldwide are using AI [11]. Despite the availability and advancement in technology, AI in deer is still underutilized due to the lack of comprehension in species-specific reproductive traits [12]. There has been an increase of interest in the female component of *R. timorensis* reproduction recently [13-18]. Concurrently, a study in stag has also been conducted with the hope to continue on the effort to move the reproduction of *R. timorensis* towards assisted reproductive technology [19]. Hence, it is vital to study semen characteristics, extension, and cryopreservation of Rusa deer semen.

The Rusa deer is a native species of Indonesia but it is the most farmed species of deer in Malaysia. Deer has long been kept in captivity, longer perhaps as hunted animals for the venison, but deer farming in Malaysia had only started in the 1980s. The first deer farm was initiated by the Department of Wildlife and National Park in Peninsular Malaysia in 1977 for the only species of deer native to Malaysia, Sambar deer (*Rusa unicolor*) [20]. Back then, demand for venison was so high at that time and Malaysia was at impending risk of overhunting wild deer by evidence of dwindling venison yield from the wild [21]. In response, 200 heads of Rusa deer was imported to Malaysia from Mauritius together with Chital deer, *Axis axis* and Sambar deer, *R. unicolor* deer from Mauritius, Sulawesi Island (Indonesia) and Sabah (East Malaysia) in 1992 [22]. Venison export value in deer industry is growing, observing more than three-fold increase of value to $221 NZD million in just over the span of 15 years in New Zealand [23]. With growing human population, there will be an increase in demand in venison and it is high time that the industry strengthens its quality and quantity of the deer population. With the reproductive biology gap being filled, it is possible to solve both needs especially by speeding up the genetic advancement of Rusa deer.

Subsequently, semen extension and cryopreservation investigations tailed to increase the dynamic and practicality of AI. Good protocol in sperm storage will allow more usage of semen through AI with knowledge of breeding season. Testosterone profile of *R. timorensis* has been determined recently and can be used as an indicator of breeding season however need to be interpreted cautiously with other compounding factors [24-27]. Semen from study animals that have died and are unable to mate due to reasons such as fracture and lameness, located in different locations from the dam can still be stored and utilized. The possibilities that could rise from AI offer potentials that could maximize the usage of studs, without actually having to keep a high number of them. Thus, AI could also be an option to reduce the cost of maintaining studs. In AI context of venison production, semen is collected from animal with desirable traits related to high productivity and quality of venison production. There is vast space to improve venison production that is yet to be fully explored.

Semen collection, characteristics, and extensions are significant to the development of AI to improve the productivity in the deer industry. However, before AI can be fully practiced, these reproductive characteristics must be established in a species-specific manner [28]. Semen extension and cryopreservation are important to speed up the genetic selection and advancement through selective and controlled breeding. Therefore, the objective of this study is to determine semen characteristics and cryopreservation of Rusa deer. The study focuses in semen characteristics that enable semen extension, cryopreservation and suggesting potential candidate for extenders to be used in Rusa deer semen.

## Materials and Methods

### Ethical approval

This research was approved by Institutional Animal Care and Use Committee Universiti Putra Malaysia, R014/2016.

### Animals

Seven healthy and matured stags were selected for this procedure from University Agricultural Park, Universiti Putra Malaysia (2.99°N, 101.7°E). The stags are all matured based on birth records, aged more than 3 years old besides a stag which was in its first antler cycle. Mean body weight of the stags is 72±1.97 kg. The stags were housed together with hinds in a herd and a semi-intensive settings. The herd is rotated every 2 months between five cyclone-fenced paddocks planted with guinea grass and supplemented with palm kernel cake every 2 days, water was given *ad libitum*.

### Semen collection

Semen collections were conducted between February 2016 and June 2016 (breeding season). Samples were collected between 2 and 3 weeks interval, collected twice for each animals. Breeding season was determined mainly based on the antler growth stage, mating and rutting behaviors and also fawning date [29]. Before semen collection, the deer were herded into a dark house to separate the stags and hinds. The stags for semen collection procedure were isolated in a different room in the dark house while the hinds were released back into the paddock. One stag was selected at a time, manually restrained and induced under general anesthesia. Drugs combination for induction used was ketamine 2 mg/kg body weight and xylazine 1.0 mg/kg body weight intravenously through the jugular vein. Semen was collected based on an established method for small wild ruminants [30]. Semen was collected using an electroejaculator (P-T Electronics, Boring, USA). The first step is to evacuate the feces from the rectum. Then, the electroejaculator probe was lubricated with KY-Jelly and gently inserted about 12 cm into the rectum. The electrode is positioned ventrally toward the pelvic floor aiming to stimulate the accessory sex glands. Stimulations were maintained at a position with positive hind limbs reflex, indicating the desired position of the probe. Electrical stimulations were applied into three series; from 1 to 6 V, restarting back from 1 to 12 V, and finally from 1 to 18 V. The stimulations were at a 3-s-on and 1-s-off rhythm, with 3 repetitions before subsequent volt increment and 1 min rest between each series. Stimulations were continued as long as seminal fluid was produced in most cases, but some stags may react differently from the electrical stimulations, and hence the decision to stop sometimes was based on the response of the stags. Erections occurred most of the time, and the semen was collected into a 20 ml sterilized falcon tube covered by aluminum foil to prevent direct sunlight and transported immediately to a mobile lab in the field. Most of the deer ejaculated at 9 V. Ejaculates contaminated with urine were discarded from the analysis. The anesthetized stag was then reversed with yohimbine (0.03 mg/kg) intravenously once the procedure is completed.

### Semen evaluation

The ejaculates were transferred using 1000 and 100 µl single-channel pipette for semen volume determination into an empty 5 ml autoclaved glass tube held in warm water bath and maintained at 37°C. Semen color was scored between 0 and 5; 0 - watery, 1 - cloudy, 2 - milky, 3 - thin creamy, 4 - creamy, and 5 - thick creamy [31]. The semen sample was then pipetted onto glass slides with eosin-nigrosin at 1:3 ratio. A smear was made onto the glass slide by using the “feathering” technique and air dried on a warm plate for morphological analysis. The mass movement and general motility were determined using a light microscope by a single technician using standard criteria in Rusa deer [19]. Mass movement was rated from 0 to 5; 0 - Dead: No motile sperm cells; 1 - Very poor: 10% motile sperm cells; 2 – Poor: No wave, 20-40% sperm cells; 3 - Fair: Small, slow moving waves, 45-65% sperm cells are active;4 – Good: Vigorous movement, wave not as rapid as score 5, 70-85% active sperm cells and 5 - Dense: Very rapid moving waves, 90% or more active sperm cells [31]. Using a drop of neat semen observed under 10× magnification, the mass movement was determined. General motility was observed under a light microscope after 1:10 dilution with normal saline, and the motility was estimated over as percent. Parallel visual general motility assessments were made by the technician and computer assisted sperm analyzer (CASA) and showed significant correlations (p<0.05) between the technician and CASA. Formal saline 1:100 was used to dilute the neat semen for concentration count with a hemocytometer. Total dilution factor for concentration count was 1:1000.

### Sperm abnormalities

Sperm abnormalities stained with eosin nigrosin were analyzed within 24 h in the laboratory based on standard guidelines [19, 32]. Sperm abnormalities count was performed on 200 spermatozoa cells. Abnormal sperm was classified into major and minor sperm defects based on the types of abnormalities. Normal sperm of Rusa deer was identified as broad and flat paddle-shaped head [19].

### Semen extension and cryopreservation

Semen collected was immediately diluted from 1:1 ratio to 1:10 depending on the initial volume of the semen. Each ejaculate (n=5) was subjected to four different types of extenders; Andromed^®^ (Andromed^®^, Minitube GmBG. Germany), BioXcell^®^ (BioXcell^®^, IMV Technologies, France), Triladyl^®^ (Triladyl^®^, Minitube GmBG. Germany) and a modified Tris-egg yolk combined with *Eurycoma longifolia* Jack [33]. The commercial extenders were prepared based on the respective manufacturers guide. Meanwhile, *E. longifolia* Jack extender was prepared in two parts; one part without glycerol for equilibration while another one with 6.4% glycerol concentration for cryopreservation. The samples were transported in a cooler box with ice packs to the laboratory and equilibrated for 4 h at 4°C in a refrigerator. 10 µl of semen will be drawn from the test tubes, thawed for 30 s at 37°C and evaluated for general motility evaluation by a single technician using light microscope. The extended semen was cryopreserved (n=3) with general motility varying from 40% to 90% based on the initial performance of the same extender during equilibration. The semen was cryopreserved using an established method [4]. The samples were thawed and analyzed again by the same technician using a light microscope. The general motility from equilibrated and cryopreserved samples was pooled based on the different types of extenders.

### Statistical analysis

Ejaculates collected were subjected to analysis using the statistical package IBM SPSS Statistics 20, values were reported as mean±standard error of mean. Analysis of variance was used to identify significant difference between ejaculates from different stages, between the collections and the various type of semen extenders at p<0.05.

## Results

### Semen evaluation

There was no significant difference (p<0.05) between characteristics of ejaculates from different stags and between collections. Therefore, the data from between collections were pooled. The results for Rusa deer semen characteristics according to volume (ml), color, sperm concentration (10^6^/ml), general motility (%), progressive motility (%), and % morphology of normal spermatozoa are 0.86±0.18, thin milky to milky, 1194.2±346.1, 82.9±2.8, 76.1±4.8, and 83.9±4.8, respectively. The seminal characteristics of Rusa deer are tabulated in Table-1.

**Table-1:** Seminal characteristics of *Rusa timorensis* deer.

Seminal characteristic	Mean±SEM	Range
Volume (ml)	0.86±0.18	0.10-2.10
Colour	Thin milky to milky	Cloudy to thick creamy
Sperm concentration (×10^6^/ml)	1194.2±346.1	94-4825
Mass movement (0-5)	2.6±0.5	0-5
General motility (%)	82.9±2.8	60-95
Progressive motility (%)	76.1±4.8	30-90
% morphology of normal spermatozoa	83.9±4.8	40.5-97.5

SEM=Standard error of mean

### Spermatozoa abnormalities

There was no significant difference (p<0.05*)* in sperm abnormalities. Spermatozoa abnormalities of Rusa deer is drawn in Table-2.

**Table-2:** Sperm abnormalities mean±SEM (%) of *Rusa timorensis*.

Sperm abormalities	Mean±SEM (%)	Range (%)
Major defects		
Acrosome defect	0.1±0.1	0-1
Dag defect	1.0±0.4	0-4
Pear-shaped effect	0.6±0.1	0-1
Decapitated sperm defect	0.1±0.1	0-0.5
Underdeveloped	0.1±0.1	0-1
Minor sperm defects		
Free normal heads	4.3±1.0	0-14
Simple bent tail	9.7±4.1	0-48

SEM=Standard error of mean

### Semen extension and cryopreservation

There was no significant difference (p<0.05) between the different types of extenders used for both; in semen extension and cryopreservation experiment. Summary of data from equilibrated and cryopreserved semen is available on Tables-3 and 4.

**Table-3:** General motility mean±SEM (%) of Rusa deer postequilibration for 4 h, at 4°C with different extenders.

Type of extender	General motility (%)	Range (%)
Andromed^®^	78.3±6.0	60-90
Triladyl^®^	70.8±8.0	45-90
BioXcell^®^	67.5±7.9	40-90
*Eurycoma longifolia* Jack	57.5±10.3	20-85

SEM=Standard error of mean

**Table-4:** General motility mean±SEM (%) of *Rusa timorensis* postcryopreservation with different extenders.

Type of extender	General motility (%)	Range (%)
Andromed^®^	53.3±10.9	40-75
Triladyl^®^	43.3±20.3	10-80
*Eurycoma longifolia* Jack	26.8±10.9	5-40
BioXcell^®^	18.3±13.3	5-45

SEM=Standard error of mean

## Discussion

Semen characteristics of Rusa deer are suitable for future work in AI. High general motility (82.9±2.8) is a good indicator that the spermatozoa will survive well enough either through chilling or cryopreservation based on our initial investigation on semen equilibration and cryopreservation results. High general motility is important in AI since cryopreservation will greatly reduce the motility. Information in AI on deer in the tropics is very limited, but the results from our study in accordance with the previous study made AI a promising venue to discover [34]. Despite early discovery of AI, the application in the industry is still at its infancy [10]. There is a dearth of literature and study of semen characteristics yet alone AI in Rusa deer. Semen characteristics of Rusa deer are now proven to be suitable for future work in AI and thus should be explored.

The challenge of working with Rusa deer semen is the minute volume of semen. However, if the work is focused in developing and improving the industry, rapid interval of collections followed by cryopreservation from selected stags may be beneficial for AI work. Successful insemination dose of deer was reported to be as low as 15×10^6^/ml progressively motile spermatozoa [35]. Based on our results, sperm concentration and volume of Rusa deer is 1194.2±346.1×10^6^/ml and 0.86±0.18 ml, respectively. Therefore in an average good quality semen, semen concentration of 900×10^6^/ml with volume 0.60 ml can be extended 60 times to a final volume yielding to 36 ml giving to 144 straws using 0.25 ml straws per animal. Overcoming the minute volume of Rusa deer semen takes into account the knowledge of semen concentration and planning of AI dose to arrive at a level of efficient AI practice.

Spermatozoa abnormalities of Rusa deer in the major defects are lower compared to the minor defects. Minor defects are moderately high on free normal heads but higher on the simple bent tail. Simple bent tail is defined as moderate unnaturally sharp angulation of the tail section [36]. This finding in Rusa deer is also consistent with the previous study in the species [19]. The simple bent tail abnormalities showed high readings in two individuals. High simple bent tail abnormalities of 48% followed by 40.5% in our case were recorded from individual #7 which was newly matured individuals in its first antler cycle. However in this study, on the second collection, the simple bent tail abnormalities were only 5.5% for individual #7. Deer is known to be infected with respiratory disease during the rutting season due to stress [37]. This could potentially interfere with the semen quality unknowingly that could contribute to the difference of semen quality. Therefore health status of the animal should always be taken into consideration and we have taken preventive measure to supplement with vitamins and prophylactic antibiotic treatment to the animal whenever deemed necessary. This study emphasizes the importance of repeated testing especially in selection of valuable breeding stag despite the early suspicion of semen quality which could be affected by many factors such as the age of the animal.

Semen abnormalities pattern can be better distinguished clearly between seasons. The difference of semen parameter was established in red deer across the breeding season [38]. For buffaloes in the sub-tropics, the pattern of simple bent tail defects across seasons is lower than Rusa deer, but the difference in season is significantly (p<0.001) higher in the rainy and winter season as compared to the summer [39]. The findings of tail abnormalities between seasons were again consistent with buffaloes in Egypt and beef bulls in Canada and showing a reduction in spermatozoa DNA integrity due to heat stress in the summer and increased in head defects in the winter [40, 41]. In general, there seems to be a tradeoff between the head and tail abnormalities within and between seasons, but more significant between seasons which could contribute from environmental changes or stress. It is hypothesized that there may be ejaculate quality changes in Rusa deer semen parameters compared between climatic seasonal changes, but more importantly between breeding and non-breeding seasons. Therefore, it is suggested that semen collection for the purpose of AI should commenced during an observed breeding behavior signifying the period of breeding season.

Tail abnormalities could also occur due to osmolality and temperature changes. This variation could be explained in the contradicting reports which showed very low tail abnormalities on various species of ruminants in the tropics through electroejaculation [30,42]. This may be due to the species-specific difference explaining the different sensitivity in osmolality, temperature changes and reaction to extenders [43,44]. However, moderate tail abnormalities are still common in other species of ruminants [4,7]. Therefore, either species-specific difference or sensitivity to osmolality and temperature changes could still not established and should be investigated further [45]. At present, there is no specific study on deer spermatozoa abnormalities in relation to fertility, however research in bull corresponds the spermatozoa abnormalities to low fertility based on outcome after AI [46]. These consistent findings of spermatozoa abnormalities in Rusa deer should be investigated further, and the simple bent tail abnormalities should be treated as true abnormalities until proven otherwise. Despite the abnormalities showed in simple bent tail, it is important to relate these findings to the clinical and practical aspect of the defects. In healthy animals, minor defects can still be tolerated as long as it does not exceed to 25% [47]. In this study, results of Rusa deer sperm defects can still be tolerated, and the tail abnormalities are the most common form of abnormalities in Rusa deer.

Despite no significant difference between the different extenders used in equilibration and cryopreservation, general motility mean pattern and consistency could still be appreciated. Semen extender performing from the best to least based on general motility was Andromed^®^, Triladyl^®^, BioXcell^®^, and Tris egg-yolk combined with *E. longifolia* Jack for equilibrated semen, while Andromed^®^, Triladyl^®^, Tris egg-yolk combined with *E. longifolia* Jack and BioXcell^®^ for cryopreserved semen. Andromed^®^ performed consistently followed by Triladyl^®^ with the best mean of general motility for both equilibrated and cryopreserved semen which is in agreement with a previous study in Iberian deer [48]. Yet a study in bull has found that the use of BioXcell^®^ in bull semen has found that it is suitable to be used in bull [49]. This propose that further investigation should be conducted to provide reason to the inefficacy in deer semen as compared to Andromed^®^. Other factor of semen quality differences could also be factor from genetic component of stags. Deer sometimes readily interbreed between species resulting in low fertility vigour hybrid [50]. Other domesticated species has developed various method to detect this which could be applicable in deer during the selection of stags [51]. This highlight the need of strict breeder selection and the role of artificial insemination in shifting the genetic exchange of the species to improve productivity. Results from this study suggested that Andromed^®^ should be considered for future work on cryopreservation for AI in Rusa deer.

The types of extenders used in deer are similar to domestic animals. The reason behind this is because the same extender used in bulls and ram performed similarly well in deer semen [10]. However, the race to find the best extender in deer continues. *E. longifolia* Jack effect as an aphrodisiac by consumption was earlier described [52]. Soybean and egg-yolk based extender has long been established in semen cryopreservation [53]. Study in *Eurycoma longifolia* has been initiated and revealed cryopreservative effect in bull spermatozoa [54]. More options to semen cryopreservation from plant material has been discovered [55]. This further reinforce the potential of an alternative plant based product to be used in semen cryopreservation. However, the usage of *E. longifolia* Jack as an extender is yet to be explored. *E. longifolia* Jack can be a candidate for a traditional option of extender used in deer besides the commercially available extender based on our results. Yet, there is still ample space for improvement since the extender performs well with bull’s semen [33]. It is obvious that the same recipe optimized for bull still need some minor modification before it can be used in deer. This creates an opportunity for locally sourced organic extender to boost the local economy since *E. longifolia* Jack is easily available in the jungle of Malaysia [56]. This is the first attempt of using *E. longifolia* Jack as an extender in deer semen.

## Conclusion

This study reports semen characteristics of Rusa deer and its plausible criteria allowing for extension and cryopreservation for future AI work. In fact, this is the first of such attempt in semen extension and cryopreservation work of Rusa deer in Malaysia. These initial findings are crucial as a foundation for future researches and development of AI of Rusa deer to improve the deer industry in the tropics.

## Recommendations

Future work should include focus in AI of Rusa deer using Andromed^®^ as an extender and improvement of *E. longifolia* Jack extender for a cheaper and readily available alternative.

## Authors’ Contributions

WNF, CAA, MRA, IDP, and FHAB carried out the experimental design, participated in the field work and preparation of the manuscript. HW, YR, FFAJ, ZAAS, and MLMA were responsible in the design, analyzing and revision of this manuscript. WH is primarily responsible for the content of this manuscript. All authors read and approved this manuscript.
